# Adipokines as Regulators of Nitric Oxide Synthase Isoforms Across Organs: Emerging Implications for Gastrointestinal Motility

**DOI:** 10.3390/ijms27188373

**Published:** 2026-09-20

**Authors:** Maria Caterina Baccari, Silvia Nistri, Elenia Cinelli, Donatella Mutolo, Eglantina Idrizaj

**Affiliations:** 1 Section of Physiological Sciences, Department of Experimental & Clinical Medicine, University of Florence, 50134 Florence, Italy; elenia.cinelli@unifi.it (E.C.); donatella.mutolo@unifi.it (D.M.); 2 Imaging Platform, Department of Experimental & Clinical Medicine, University of Florence, 50139 Florence, Italy; silvia.nistri@unifi.it

**Keywords:** adipokines, nitric oxide synthase, nitrergic neurotransmission, gastrointestinal motility, enteric nervous system, leptin, adiponectin, resistin

## Abstract

Adipose tissue secretes bioactive mediators known as adipokines. Beyond their well-established roles in energy homeostasis, appetite regulation, insulin sensitivity, and inflammation, adipokines have emerged as important modulators of inter-organ communication, influencing cardiovascular, neural, immune, and respiratory functions. Growing evidence indicates that many of these effects involve nitric oxide (NO) signaling. NO is produced by three major nitric oxide synthase (NOS) isoforms: neuronal (nNOS), endothelial (eNOS), and inducible (iNOS). Experimental evidence indicates that leptin, adiponectin, and resistin can modulate NOS expression or activity and NO bioavailability in a tissue- and context-dependent manner. In the gastrointestinal tract, these adipokines can influence nitrergic signaling and motility in *ex vivo* rodent preparations. In this review, we compare leptin–, adiponectin–, and resistin–NOS interactions across multiple organs, evaluating direct findings and their possible involvement in enteric nitrergic neurotransmission and gastrointestinal motor regulation. We highlight an emerging three-way regulatory pattern in which leptin appears to exert context-dependent actions, adiponectin predominantly preserves constitutive nitrergic signaling, and resistin shows acute versus chronic effects that may depend on exposure duration. Although direct enteric evidence remains limited and human studies are needed, a better understanding of adipokine-dependent NOS regulation might provide potential therapeutic approaches for gastrointestinal dysmotility.

## 1. Introduction

Adipose tissue has increasingly emerged from a passive energy storage compartment to an endocrine organ capable of secreting numerous biologically active mediators collectively known as adipokines [1]. These molecules, initially mainly recognized in the context of appetite regulation, insulin sensitivity, and inflammation, are now recognized as pleiotropic regulators of inter-organ communication that modulate vascular tone, immune activation, redox signaling, and neuronal function, acting as integrators of metabolic and tissue homeostasis [1,2,3,4,5,6].

Notably, several adipokines converge on nitric oxide (NO) signaling pathways. NO is synthesized from L-arginine via nitric oxide synthases (NOS), which exist in three main isoforms: the neuronal NOS (nNOS), the endothelial NOS (eNOS), and the inducible NOS (iNOS) [7,8,9,10,11]. Constitutive isoforms (nNOS, eNOS) are Ca^2+^-dependent and generate physiological NO levels, while iNOS is mainly induced by inflammatory stimuli and produces high and sustained NO levels [12,13]. The NOS system integrates metabolic, inflammatory, and redox signals, with functional output determined by transcriptional regulation, post-translational modifications, substrate availability, and oxidative stress [14,15]. An additional level of NOS regulation relevant to metabolic and inflammatory conditions is enzyme coupling. Under physiological conditions, efficient NOS activity requires adequate availability of L-arginine and essential cofactors, particularly tetrahydrobiopterin (BH4), allowing electron transfer to remain coupled to NO synthesis [16]. When BH4 availability is reduced or compromised by oxidation, NOS becomes uncoupled and preferentially generates superoxide over NO. This process further decreases NO bioavailability and exacerbates oxidative and nitrosative stress, promoting peroxynitrite formation and thereby amplifying oxidative damage and additional NOS dysfunction [16]. Alterations in substrate availability, arginase activity, and the cellular redox environment may therefore influence the balance between coupled physiological NO production and uncoupled oxidative signaling [17]. NOS activity is also regulated by site-specific phosphorylation. For eNOS, Ser1177 phosphorylation, promoted by kinases such as Akt and AMPK, enhances enzymatic activity, whereas Thr495 phosphorylation reduces calmodulin binding and inhibitory regulation [9,18]. Additional regulatory sites, including Ser633, may contribute to the maintenance of eNOS activity and sustained NO production depending on the cellular and stimulus context [9]. nNOS activity is likewise regulated by phosphorylation-dependent mechanisms involving several kinases, although the functional consequences are highly cell- and stimulus-dependent [18]. Therefore, references in this review to “phosphorylation-dependent NOS regulation” indicate specific molecular regulatory events rather than a generic increase or decrease in NO production. Importantly, the extent to which individual adipokines directly regulate these phosphorylation events in enteric tissues remains poorly defined compared to cardiovascular and other systems. Subcellular localization of NOS represents a further determinant of its function because the spatial organization of the enzyme influences its access to signaling partners, Ca^2+^-dependent regulatory mechanisms, and local redox conditions [19]. In particular, eNOS activity is closely linked to its membrane-associated localization within specialized cellular domains, whereas changes in intracellular distribution may modify the efficiency and spatial specificity of NO signaling [20]. Direct evidence that adipokines regulate NOS localization remains limited and largely derived from non-gastrointestinal experimental systems. Accordingly, intracellular localization is considered in this review as a potential regulatory level rather than as a fully validated mechanism common to all adipokines.

Adipokine-dependent regulation of NOS pathways has been documented in several systems, including the cardiovascular, immune, reproductive, respiratory, and nervous systems [21,22,23,24,25,26]. Emerging evidence suggests that a similar adipokine–NOS regulatory network also operates within the gastrointestinal tract [27,28]. This represents a particularly relevant target for adipokine-dependent NOS regulation because, besides playing key roles in mucosal blood flow, epithelial barrier integrity, secretion, and immune homeostasis, NO is one of the principal enteric inhibitory neurotransmitters responsible for smooth muscle relaxation [29,30,31,32,33].

In the gastrointestinal tract, nNOS represents the principal source of inhibitory neuronal NO, whereas eNOS contributes to mucosal perfusion and vascular homeostasis. In contrast, iNOS is minimally expressed under physiological conditions but is strongly induced during inflammation, leading to sustained NO production and impaired neuroeffector function [34]. Accordingly, disturbances in NOS expression or NO bioavailability are implicated in several gastrointestinal disorders, including functional dyspepsia, gastroparesis, irritable bowel syndrome, chronic intestinal pseudo-obstruction, postoperative ileus, inflammatory bowel disease, diabetic enteropathy, and obesity-associated intestinal inflammation [13,35,36,37,38,39]. Of note, gastrointestinal dysmotility may result from disruption in the balance among nNOS/eNOS activity, iNOS induction, oxidative stress, and NOS uncoupling, especially in obesity and metabolic disorders [40].

Among the numerous adipokines identified to date, leptin, adiponectin, and resistin are the most representative for understanding how metabolic signals regulate constitutive and inducible NOS isoforms under both physiological and pathological conditions [21,41,42]. Additional adipokines, including apelin, chemerin, visfatin, and omentin, as well as inflammatory cytokines such as TNF-α and IL-6, have been implicated in the regulation of NO signaling [43] as well as in gastrointestinal physiology and pathophysiology [44,45,46,47,48]. In particular, apelin inhibits gastric motor functions in rats [46,47]. However, these mediators are not discussed in detail here because, compared with leptin, adiponectin, and resistin, their specific effects on NOS and their contribution to enteric nitrergic regulation remain poorly characterized, or not characterized at all. Therefore, in the present review, we focus on adipokines for which the available evidence on NOS-related mechanisms and gastrointestinal nitrergic regulation yields a sufficiently robust framework for meaningful integration and comparative analysis.

**Figure 1:**
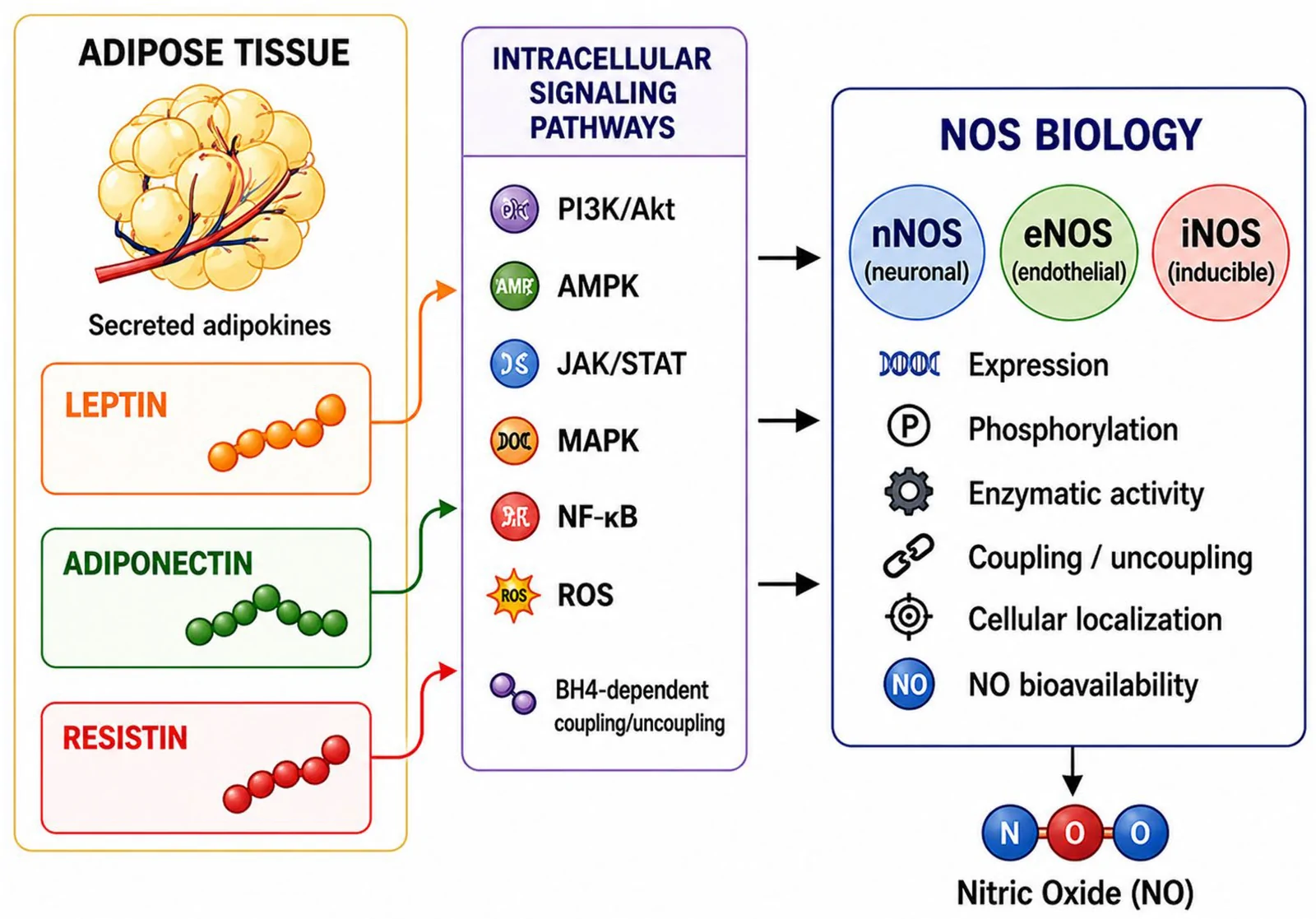
Modulation of NOS biology by adipokines. Leptin, adiponectin, and resistin have been reported to influence multiple levels of NOS/NO signaling, although the evidence supporting each regulatory mechanism differs among adipokines, tissues, and experimental models. Through signaling pathways including PI3K/Akt, AMPK, JAK/STAT, MAPK, and NF-κB, adipokines may influence NOS isoform expression, phosphorylation-dependent regulation, enzymatic activity, oxidative environment, coupling efficiency, and NO bioavailability. Some of these regulatory levels are directly supported by experimental evidence in specific tissues, whereas others represent proposed pathways.

Despite exerting distinct biological functions, leptin, adiponectin, and resistin influence NOS signaling through pathways including PI3K/Akt, AMPK, MAPK, JAK/STAT, and NF-κB [49], as illustrated in Figure 1. Available evidence indicates that these adipokines can influence multiple levels of NOS/NO signaling, including NOS expression, enzymatic activity, phosphorylation-dependent regulation, redox balance, and NO bioavailability. However, the nature and strength of these effects vary substantially across adipokines, tissues, cell types, and experimental models. Thus, although their actions may extend beyond simple changes in NO output, a unified role as direct regulators of all aspects of NOS biology should currently be regarded as a conceptual framework rather than an established mechanism. Accordingly, in this review, the term “NOS regulation” is used only where evidence supports changes in NOS expression, activity, or relevant regulatory mechanisms, distinguishing these effects from changes in NO output alone.

Therefore, this review focuses on leptin, adiponectin, and resistin as the most representative adipokines whose interactions with NOS signaling have been extensively characterized in several tissues, organs, and systems, including cardiovascular, neuronal, immune, metabolic, reproductive, and respiratory systems, and are now increasingly recognized in gastrointestinal physiology and pathophysiology. We highlight here the specific adipokines that influence gastrointestinal motility through NOS interactions. To this end, particular attention is given to their ability to modulate nNOS, eNOS, and iNOS pathways and to the emerging role of adipokine-dependent nitrergic regulation in enteric neuromuscular function and gastrointestinal motility, suggesting that adipokine-dependent modulation of NOS pathways reflects a broad biological mechanism operating across different physiological systems.

While our previous reviews [28,29] established main concepts regarding adipokine actions, the present work offers a distinct incremental contribution by providing a critical three-way comparison of the directional models of leptin, adiponectin, and resistin as regulators of NOS/NO signaling across distinct organ systems, with particular emphasis on the acute versus chronic distinction for resistin, and the evidence gap between enteric animal models and human gastrointestinal diseases.

The following sections summarize current knowledge on the interactions between leptin, adiponectin, and resistin and NOS signaling across different systems, highlighting their increasingly recognized contribution to the regulation of gastrointestinal motility.

## 2. Literature Search Strategy

The literature search was last updated in September 2026. Searches were conducted in PubMed, Scopus, and Web of Science using combinations of the following keywords and Boolean operators: “adipokine” OR “leptin” OR “adiponectin” OR “resistin” AND “nitric oxide” OR “nitric oxide synthase” OR “NOS” AND organ- or gastrointestinal-related terms, when relevant, such as “central nervous system”, “cardiovascular”, “immune”, “respiratory”, “gastric motility”, “intestinal motility”, “enteric neurons”, “enteric neurotransmission”, “nitrergic neurotransmission”, “enteric nervous system”, “smooth muscle”, “interstitial cells of Cajal”. Search inclusion criteria encompassed English-language original research articles, experimental studies (*in vitro*, *ex vivo*, and *in vivo*), and relevant review articles addressing leptin, adiponectin, or resistin in relation to NO/NOS signaling, with particular emphasis on gastrointestinal physiology and motility. Exclusion criteria comprised publications not addressing these adipokines or NO/NOS-related mechanisms, studies lacking relevance to the biological systems considered in this review, and non-English publications. Particular attention was paid to literature published during the past 10 years, from 2016 to 2026, while seminal earlier studies were retained when they provided essential or historical evidence. As this is a narrative review, the search was not intended as a systematic or exhaustive evidence synthesis.

## 3. Adipokine–NOS Interactions

### 3.1. Leptin as Modulator of NOS Isoforms

Leptin is a 16 kDa peptide hormone predominantly secreted by white adipose tissue and encoded by the obesity (ob) gene. Since its discovery in 1994, leptin has been recognized as a key regulator of energy balance, linking peripheral energy stores to central neuroendocrine circuits that control appetite and metabolism [50,51,52,53].

Circulating leptin concentrations generally correlate with adipose tissue mass and nutritional status, acting as a long-term signal of energy availability: following its release into the circulation, leptin crosses the blood–brain barrier and binds to leptin receptors (LEP-R) expressed in several hypothalamic nuclei, including the arcuate, paraventricular, ventromedial, and dorsomedial nuclei, where it suppresses food intake and promotes energy expenditure [51,54,55].

The leptin receptor belongs to the class I cytokine receptor superfamily and exists in several alternatively spliced isoforms. Among these, the long isoform LEP-Rb represents the principal signaling receptor responsible for most metabolic and neuroendocrine effects of leptin, through the activation of multiple intracellular pathways [55,56,57].

Among adipokines, leptin is one of the most extensively studied in relation to NO signaling. Increasing evidence indicates that leptin modulates all three NOS isoforms through PI3K/Akt-, JAK/STAT-, MAPK-, and NF-κB-dependent mechanisms, thereby influencing cardiovascular, neuronal, immune, metabolic, reproductive, and respiratory functions [21,23,24,41,58].

Leptin–NOS interactions have been documented in the central nervous system, where leptin has been shown to modulate nNOS activity and NO production within discrete hypothalamic regions, where NO may influence neuronal excitability, synaptic plasticity, and neuroendocrine output [26]. Within hypothalamic circuits, nNOS-derived NO participates in the integration of metabolic signals controlling feeding behavior, autonomic activity, and energy expenditure [23,41].

Among peripheral systems, the best characterized model of leptin–NOS interaction is represented by the cardiovascular system. Under physiological conditions, leptin stimulates endothelial NO production through activation of the PI3K/Akt/eNOS pathway, resulting in increased eNOS phosphorylation, enhanced NO bioavailability, and improved endothelium-dependent vasodilation [21,59]. These actions contribute to vascular homeostasis, blood pressure regulation, maintenance of endothelial integrity, and inhibition of platelet aggregation [9,21]. Human recombinant leptin has also been shown to activate eNOS in cultured endothelial cells, further supporting a direct role of leptin in endothelial NO regulation [60]. Endothelial dysfunction, oxidative stress, reduced NO bioavailability, and NOS uncoupling are associated with chronic hyperleptinemia and leptin resistance in obesity [24,59,61,62]. These alterations contribute to vascular remodeling, atherosclerotic progression, and impaired metabolic regulation [24,59,63,64]. Leptin also stimulates fatty acid oxidation, glucose utilization, mitochondrial function, and insulin sensitivity through mechanisms partially involving constitutive NOS activation and increased NO bioavailability [41,65]. Alterations of this pathway have been implicated in obesity-associated insulin resistance and metabolic dysfunction [64].

Leptin–NOS interactions are also relevant in the reproductive system, where they lead to increased NO production, which participates in hypothalamic-pituitary-gonadal axis regulation and reproductive maturation: leptin deficiency results in infertility and delayed puberty, whereas leptin replacement restores reproductive competence in both experimental models and humans [23,66]. Leptin has further been reported to induce uterine relaxation in pregnant mice through eNOS expression and activation [67].

Among the major NOS isoforms, both constitutive and inducible NOS pathways have been reported to be activated by leptin in the respiratory system. Indeed, leptin receptors are expressed in airway epithelial cells, alveolar macrophages, and pulmonary endothelial cells. Leptin/obR signaling promotes the polarization of pulmonary macrophages towards the M1 inflammatory phenotype by activating the JNK/STAT3/AKT signaling pathway and iNOS in obesity-associated asthma [68], increasing NO production in cultured bovine pulmonary endothelial cells [69].

A correlation among serum levels of leptin, leptin receptors, and NO has been reported in patients with obstructive sleep apnea syndrome, where the key feature is chronic intermittent hypoxia [70].

Notably, in the liver, the effects of chronic hyperleptinemia have been predominantly associated with iNOS induction. In fact, while physiological leptin signaling participates in the regulation of hepatic lipid metabolism and insulin sensitivity, chronic hyperleptinemia promotes inflammatory cytokine release, Kupffer cell activation, oxidative stress, and iNOS induction, mechanisms implicated in the progression of Non-Alcoholic Fatty Liver Disease (NAFLD), steatohepatitis, and liver fibrosis [71,72].

Increased iNOS expression with inflammatory NO generation has also been reported to be induced by leptin in macrophages and other immune cells, thereby promoting inflammatory signaling, cytokine production, and NF-κB activation [13,24,73,74].

Taken together, the available evidence indicates that leptin regulates NOS signaling in a context-dependent manner, promoting constitutive NO production under physiological conditions and favoring inflammatory NOS activation during metabolic dysfunction.

#### Leptin as a Modulator of NOS Signaling in the Gastrointestinal Tract

Along the gastrointestinal tract, leptin receptors are expressed in the gastric mucosa, vagal afferents, enteric neurons, and smooth muscle layers, indicating that leptin can directly influence gastrointestinal functions [75,76]. Increasing evidence from gut–brain axis studies further supports the existence of bidirectional neuro-metabolic circuits linking adipose tissue, central energy sensing, and enteric motor regulation [77]. Interestingly, the stomach has been reported as an extra-adipose source of leptin in both rodents and humans [78,79,80].

Experimental studies demonstrate that leptin delays gastric emptying, reduces spontaneous contractility, and regulates intestinal transit [75,81,82]. In the isolated rat proximal colon, leptin alters enteric neurotransmission and motor responses, exerting effects on both cholinergic and nitrergic pathways [27]. Preclinical evidence also indicates an interaction between leptin signaling and enteric nitrergic function. In rats, kaolinite-induced changes in enteric responses were associated with alterations in leptin-sensitive and NOS-dependent neurotransmission, while in mice, diet-induced obesity was associated with preservation of gastric nitrergic neurons and increased leptin/GDNF signaling [83,84]. These findings suggest that leptin modulates enteric nitrergic function, although a direct regulation of enteric NOS isoforms remains to be established.

In pathological conditions, such as obesity and metabolic syndrome, chronic hyperleptinemia is associated with leptin resistance and systemic oxidative/inflammatory alterations [55,85,86]. However, whether these changes impair enteric nNOS/eNOS signaling or upregulate enteric iNOS expression remains unproven, leaving the direct link to obesity-associated gastrointestinal dysmotility hypothetical.

In the gastrointestinal tract, impaired leptin responsiveness may therefore blunt constitutive nNOS/eNOS activation, while simultaneously promoting NF-κB-dependent inflammatory signaling and iNOS overexpression. This pathological shift alters the quality of NO signaling, replacing the release of NO as a neurotransmitter with its sustained production, leading to inflammation and oxidative nitrergic instability.

### 3.2. Adiponectin as Modulator of NOS Isoforms

Adiponectin is one of the most abundant circulating adipokines secreted by white adipose tissue. Structurally, adiponectin belongs to the complement C1q protein family, and circulates in several oligomeric forms, including low-molecular-weight trimers, middle-molecular-weight hexamers, and high-molecular-weight multimers [87]. These isoforms exhibit distinct biological activities and tissue-specific effects, with high-molecular-weight adiponectin generally considered the most metabolically active form [88,89]. Unlike most adipose-derived molecules, adiponectin’s plasma levels are inversely correlated with adiposity, declining significantly in obesity, metabolic syndrome, type 2 diabetes mellitus, and cardiovascular disease [3,90,91].

The biological actions of adiponectin are primarily mediated through two seven-transmembrane receptors, AdipoR1 and AdipoR2, which display distinct tissue distributions and signaling properties, and activate AMPK- and PPARα-dependent pathways involved in metabolic regulation and oxidative stress control [89,92,93,94,95]. Through activation of the AMPK pathway, adiponectin promotes insulin sensitivity, fatty acid oxidation, mitochondrial function, and anti-inflammatory responses while reducing oxidative stress [3,90,96,97,98]. In addition, adiponectin interacts with T-cadherin, a glycosylphosphatidylinositol-anchored receptor, which contributes to tissue-specific adiponectin signaling and cardiovascular protection [99,100,101].

Adiponectin receptors are widely expressed within the central nervous system, including the hypothalamus, hippocampus, cortex, and brainstem [97,98]. Experimental studies suggest that adiponectin contributes to neuroprotection by limiting oxidative stress, mitochondrial dysfunction, neuroinflammation, and neurodegenerative processes, while supporting physiological nitrergic signaling by preserving constitutive eNOS and nNOS activity, and inhibiting iNOS expression [102,103,104,105,106]. Adiponectin deficiency is associated with impaired neurogenesis, neuronal insulin resistance, and the exacerbation of pathological hallmarks of Alzheimer’s disease [107].

Peripheral adiponectin–NOS interactions have also been reported, and the cardiovascular system represents the best-characterized model. Adiponectin stimulates endothelial NO production through activation of AMPK-dependent eNOS phosphorylation, increasing NO bioavailability, and preserving endothelial homeostasis [3,22,49,108,109]. In addition to directly enhancing eNOS activity, adiponectin limits ROS generation, reduces oxidative degradation of NO, and prevents eNOS uncoupling, thereby preserving endothelial homeostasis and vascular function [25,94,108,110]. By maintaining physiological NO signaling, adiponectin contributes to anti-atherogenic protection, inhibition of vascular inflammation, preservation of endothelial barrier integrity, and improvement of insulin-mediated vascular responses [3,22,63,111]. Reduced adiponectin levels in obesity are therefore strongly associated with endothelial dysfunction and impaired NO bioavailability. Cardiac tissues represent an important target of adiponectin-dependent NO regulation. Experimental studies have demonstrated that adiponectin protects against ischemia–reperfusion injury, oxidative stress, and inflammatory damage through mechanisms involving AMPK activation and preservation of eNOS signaling [3,96,112,113]. In metabolic tissues, adiponectin enhances glucose uptake, fatty acid oxidation, mitochondrial biogenesis, and insulin sensitivity [3,90]. Beyond its metabolic actions, adiponectin exerts potent anti-inflammatory effects by suppressing NF-κB activation, reducing cytokine production, and limiting oxidative stress [3,94,96]. Through these mechanisms, adiponectin also attenuates pathological iNOS induction in immune cells, contributing to the maintenance of physiological NO homeostasis during inflammatory conditions [3].

Protective adiponectin–NOS interactions have additionally been reported in pulmonary tissues, where adiponectin preserves NO bioavailability, limits oxidative stress, improves endothelial function, and attenuates inflammatory responses within pulmonary vascular and airway compartments [3]. Interestingly, adiponectin has been shown to inhibit the expression of iNOS and NO overproduction in mice with obesity-related asthma [114].

Taken together, the available evidence supports the concept that adiponectin promotes physiological nitrergic signaling through the enhancement of constitutive NOS activity, the preservation of NO bioavailability, the reduction in oxidative stress, and the suppression of pathological iNOS induction across multiple organs and systems.

#### Adiponectin as a Modulator of NOS Signaling in the Gastrointestinal Tract

Adiponectin receptor expression has been demonstrated throughout the gastrointestinal tract of rodents and humans [115,116,117,118], and its production has also been reported at the gastric level in rodents [119].

In isolated mouse gastric fundus preparations, adiponectin reduces neurally evoked contractile responses while potentiating inhibitory responses [116,120,121]. These effects are abolished by NOS blockade, indicating a close dependence on nitrergic neurotransmission, confirmed by the ability of the hormone to upregulate nNOS expression and increase the percentage of nNOS-positive neurons in the myenteric plexus [122].

Compelling data indicate that adiponectin may act directly on the enteric neuromuscular compartment via AdipoR1- and AMPK-dependent mechanisms, promoting smooth muscle relaxation independently of neuronal influence [121]. More recent work also shows that adiponectin modulates murine gastric smooth muscle cell excitability and contractility via sphingosine kinase 2/S1P signaling, providing an additional pathway parallel to classic AMPK activation [123].

A role for enteric glia in adiponectin-dependent regulation of ileal motility has been further proposed [117]. The increasing recognition of enteric glia as active regulators of inhibitory neurotransmission [32] indeed raises the possibility that adiponectin-dependent neuro-glial interactions represent an additional regulatory layer modulating constitutive nitrergic signaling. In this context, adiponectin has been reported to depress mouse ileal contractility through mechanisms likely involving AdipoR1 expressed on enteric glial cells and subsequent NOS interaction [117], suggesting a possible physiological link between this hormone and the ileal brake mechanism [124].

All the above findings support a role for adiponectin in facilitating enteric nitrergic neurotransmission and maintaining coordinated motor activity. In this view, gastroprotective effects involving NO-dependent pathways have also been recently reported in experimental models of gastric injury [125]. Moreover, reduced adiponectin levels in obesity may contribute to impaired inhibitory neurotransmission, decreased NO bioavailability, and enteric neuromotor dysfunction.

The NO-mediated effects of adiponectin on gastrointestinal motility have been hypothesized to represent peripheral signals involved in the regulation of food intake through the gut–brain axis [117,126,127], thereby contributing to its central anorexigenic actions reported in rodents [128]. Notably, the gastrointestinal tract plays a pivotal role in the regulation of food intake and energy homeostasis by integrating hormonal, neural, and mechanical signals [81,129]. Additional evidence supporting the involvement of adiponectin in gut–brain communication comes from studies showing the expression of adiponectin receptors on vagal afferents of the mouse gastric antrum [119]. These findings suggest that adiponectin may modulate vagal sensory pathways involved in the transmission of gastrointestinal signals to the central nervous system.

Taken together, the available gastrointestinal literature reviewed here identifies adiponectin as having the most fully characterized role in constitutive nitrergic modulation, particularly in mouse gastric fundus and ileal preparations.

### 3.3. Resistin as Modulator of NOS Isoforms

Resistin is a cysteine-rich secretory protein whose cellular source differs markedly between species: in rodents it is predominantly adipocyte-derived [130], but it is also expressed in multiple other structures, including the hypothalamus [131] and gastrointestinal tract [132], whereas in humans, it is produced mainly by monocytes/macrophages [133].

In contrast to leptin and adiponectin, resistin is primarily associated with inflammatory responses and chronic metabolic dysfunctions. Elevated circulating resistin levels have been linked to obesity, insulin resistance, type 2 diabetes mellitus, metabolic syndrome, atherosclerosis, and cardiovascular disease [109,134,135].

Although its signaling mechanisms remain incompletely defined, resistin has been reported to activate Toll-like receptor 4 (TLR4)- and adenylate cyclase-associated protein 1 (CAP1)- dependent pathways involving NF-κB, JNK, p38 MAPK, and cAMP signaling cascades [133,136]. Activation of these pathways promotes inflammatory gene transcription, oxidative stress generation, and endothelial dysfunction.

Increasing evidence indicates that resistin exerts significant actions within the central nervous system, contributing to neuroinflammation. Experimental studies have demonstrated increased blood–brain barrier permeability, microglial activation, cytokine production, and oxidative stress following resistin exposure [137,138]. These effects have been associated with obesity-related hypothalamic inflammation, altered central metabolic regulation, and neuroinflammatory processes [137,138,139,140]. However, direct evidence regarding the effects of resistin on specific NOS isoforms within the central nervous system remains limited. In particular, the involvement of iNOS or alterations in constitutive nitrergic signaling have not been directly established in the brain. Evidence from peripheral inflammatory tissues indicates that resistin can modulate NO/NOS signaling [110,141,142]; therefore, a potential role for resistin in the central nervous system nitrergic remodeling may be hypothesized, but remains to be experimentally demonstrated.

The cardiovascular system provides some of the strongest evidence for resistin-dependent modulation of NOS signaling. Experimental studies have shown that resistin reduces eNOS expression and activity, impairs Akt-dependent eNOS phosphorylation, increases ROS generation, and decreases NO bioavailability [134,143,144]. These alterations contribute to endothelial dysfunction, vascular inflammation, and progressive vascular remodeling [134,143]. Consequently, resistin is generally considered a negative regulator of constitutive eNOS signaling and an important contributor to obesity-associated cardiovascular risk [134].

Resistin also plays a central role in the interaction between metabolism and innate immunity. In macrophages and other immune cells, resistin promotes inflammatory cytokine production and NF-κB-dependent responses [133,145].

Although no direct evidence exists on resistin–NOS interactions in the respiratory system, the contribution of resistin to the initiation and progression of lung diseases, such as pulmonary hypertension, asthma/allergic airway inflammation, chronic obstructive pulmonary disease, and fibrosis, has been reported [146]. In particular, resistin-like molecules (RELMβ) may contribute to hypoxia-induced pulmonary vascular remodeling processes or hypoxia-related fibrotic lung disease, while also playing an important role in allergic disease in human and mouse lungs [147].

Taken together, the available evidence identifies resistin as a pro-inflammatory regulator of NOS biology. In contrast to adiponectin, which primarily preserves constitutive NOS signaling, resistin generally promotes suppression of eNOS-dependent pathways, induction of iNOS activity, oxidative stress generation, and inflammation. Consequently, chronic resistin elevation appears to favor the transition from physiological nitrergic signaling toward pathological nitrergic remodeling across multiple organs and systems.

#### Resistin as a Modulator of NOS Signaling in the Gastrointestinal Tract

Resistin expression has been revealed in the gastrointestinal tract of rodents [132] and available evidence indicates that resistin exerts complex effects on enteric nitrergic regulation. At present, few studies have addressed the resistin–NOS interaction under physiological conditions. However, some experimental studies performed in gastric preparations from mice demonstrated that acute resistin exposure attenuates excitatory contractility and enhances neurally mediated inhibitory responses, effects that disappear after NOS inhibition. Furthermore, resistin has been shown to upregulate nNOS expression and to increase the percentage of nNOS-positive neurons in the myenteric plexus, suggesting that it may acutely facilitate enteric inhibitory nitrergic neurotransmission under selected physiological conditions [148]. Interestingly, co-treatment with adiponectin and resistin did not induce additional effects in gastric preparations, suggesting that both adipokines may share nNOS upregulation as a common target, likely representing a dual physiological control mechanism that ensures gastric fundus relaxation [122].

Although these findings seem in contrast with the predominantly pro-inflammatory actions reported for resistin in other tissues, differences between acute and chronic exposure provide a plausible explanation. Chronic elevation of resistin triggers NF-κB activation, cytokine release, oxidative stress, and inflammatory remodeling, processes known to induce iNOS expression and impair constitutive NO signaling across several tissues [42]. Whether a similar mechanism operates in the gastrointestinal tract remains to be directly established. Nevertheless, chronic resistin exposure could alter the balance between constitutive and inflammatory NO signaling, possibly through increased oxidative stress and ROS-mediated NO inactivation. It may therefore be speculated that, within the enteric environment, prolonged exposure to resistin could contribute to impaired inhibitory neurotransmission, neuroinflammation, and dysmotility. The final functional outcome is likely influenced by exposure duration, inflammatory status, and local oxidative conditions [149,150,151].

## 4. Comparative Overview of Adipokine–NOS Interactions in the Gastrointestinal Tract

Although leptin, adiponectin, and resistin participate in the regulation of gastrointestinal physiology, their interactions with NO/NOS signaling appear to differ substantially in terms of experimental evidence, target cells, NOS isoforms, and temporal context. Moreover, findings within the gastrointestinal tract do not always mirror observations reported in other systems, including the nervous, cardiovascular, metabolic, immune, and respiratory systems. Therefore, the available data support a model of adipokine-dependent modulation of gastrointestinal nitrergic function.

Leptin primarily acts as a context-dependent modulator of nitrergic signaling. In the gastrointestinal tract, its effects on motility are experimentally supported, whereas the proposed regulation of enteric nNOS/eNOS and the existence of an obesity-associated pathological switch remain inferential.

Adiponectin exhibits the most developed direct gastrointestinal evidence for preservation or enhancement of constitutive nitrergic responses in the examined models. Experimental evidence indicates that adiponectin enhances inhibitory neurotransmission, promotes nNOS expression, preserves NO bioavailability, and supports coordinated motor activity through neuronal, glial, and muscular mechanisms. A role of adiponectin in inflammatory bowel disease has also been reported [152]; however, these inflammatory effects should be considered separately from the NOS-dependent motor mechanisms.

Resistin displays a more complex profile. Acute exposure may enhance inhibitory nitrergic responses in the mouse gastric fundus, whereas chronic elevation is associated with inflammatory activation and conditions favoring iNOS induction in non-gastrointestinal tissues. The chronic gastrointestinal consequence remains hypothetical and may depend strongly on the duration of exposure.

**Figure 2:**
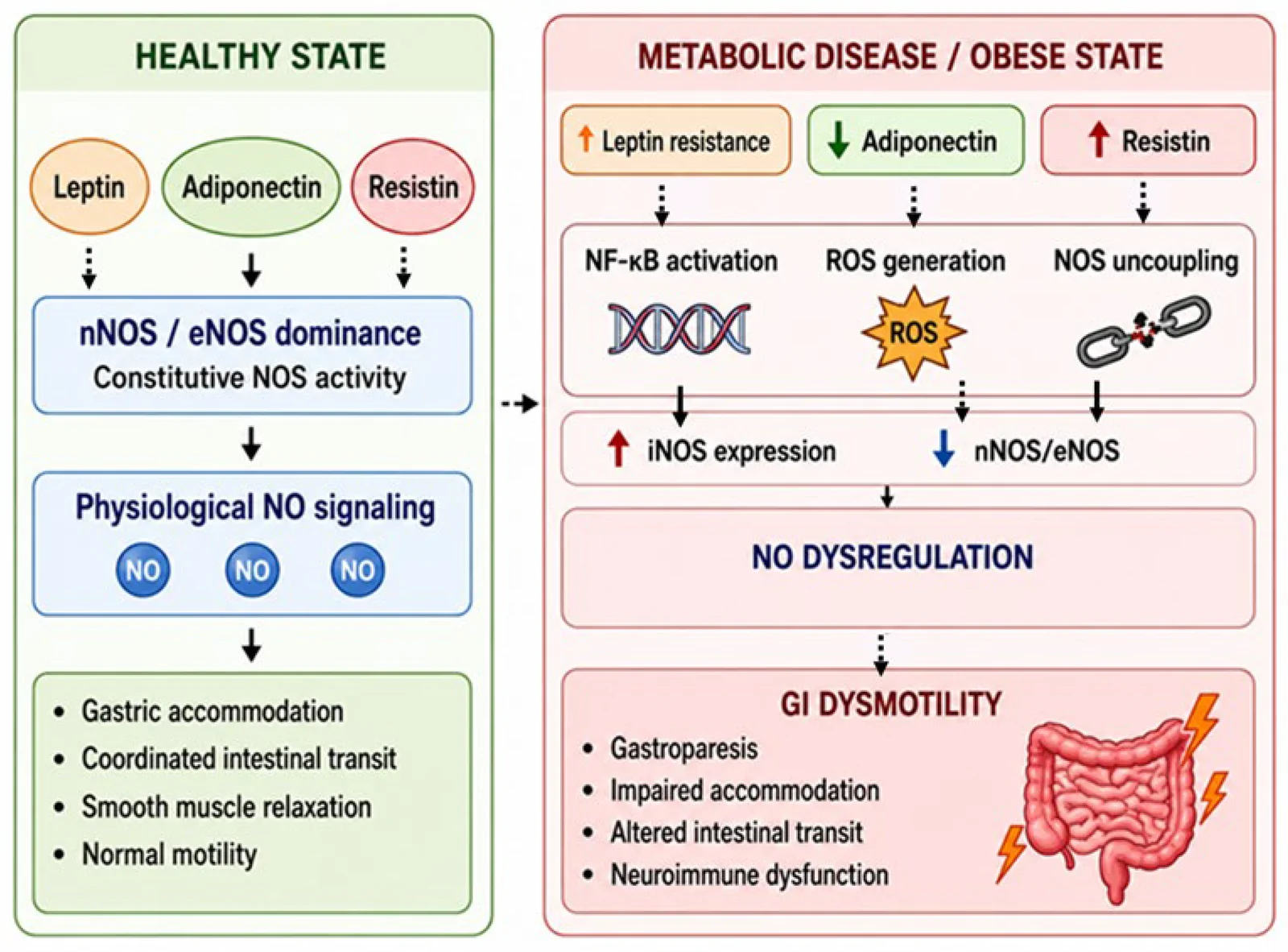
Adipokine–NOS interactions in gastrointestinal physiology and pathophysiology. Solid arrows indicate relationships supported by direct experimental evidence, whereas dashed arrows indicate proposed, inferred, or extrapolated relationships. Under physiological conditions, constitutive NOS-derived NO signaling contributes to gastrointestinal motor functions. In metabolic disease and obesity, alterations in adipokine profiles may interact with inflammatory and oxidative pathways and contribute to altered NOS/NO signaling. The complete sequence linking adipokine alterations to gastrointestinal dysmotility represents a biologically plausible framework that requires further direct validation, particularly in human gastrointestinal tissues.

These observations suggest that adipokines may influence gastrointestinal nitrergic signaling at multiple levels, including NO availability, NOS-related pathways, and the redox environment; however, direct evidence for regulation of the balance among NOS isoforms remains limited and adipokine-specific. Since gastrointestinal motility critically depends on tightly regulated inhibitory neurotransmission, disturbances in this balance may have important functional consequences, as illustrated in Figure 2. This concept is further supported by evidence demonstrating that several gut-derived hormones and neuroendocrine mediators, including other regulatory peptides, are also capable of modulating gastrointestinal motility through regulation of NOS isoforms and nitrergic neurotransmission [116,153,154,155,156].

Importantly, adipokine-dependent modulation of NOS pathways is unlikely to be restricted to enteric neurons. Endothelial cells, smooth muscle cells, enteric glia, interstitial cells of Cajal (ICC), and resident immune cells may all contribute to an integrated neuroimmune-metabolic network linking systemic energy status to gastrointestinal function [157,158]. ICC function depends on specialized ion channels and Ca^2+^ handling mechanisms that regulate cellular excitability and gastrointestinal pacemaker activity. In particular, Anoctamin 1 (ANO1), a Ca^2+^-activated Cl^−^ channel highly expressed in ICC, plays a central role in the generation and propagation of slow-wave activity, whereas the transient receptor potential melastatin-type 7 (TRPM7) channel is involved in the electrical activity and pacemaker function of gastrointestinal ICC [158,159]. However, despite the biological relevance of ICC and these ionic mechanisms to gastrointestinal motility, direct evidence demonstrating that leptin, adiponectin, or resistin specifically modulate ANO1 or TRPM7 expression or function in gastrointestinal ICC remains limited [158,159]. Therefore, a direct adipokine–ion channel–ICC mechanism remains unproven. This represents a critical knowledge gap since adipokine-dependent changes in neuronal, glial, vascular, inflammatory, or smooth muscle signaling may indirectly affect ICC function, thereby contributing to altered gastrointestinal motor behavior. In particular, gastrointestinal motility is regulated by a highly integrated neuroeffector network involving smooth muscle cells, enteric neurons, ICC, enteric glia, immune cells, and extrinsic autonomic inputs [32,158,160,161,162,163,164]. Adipokine signaling may interact with components of this network and modulate gastrointestinal motor function. However, the available evidence remains largely experimental and context-dependent, and direct effects on specific cellular components of the enteric neuromuscular system have not been established for all adipokines. Accordingly, therapeutic targeting of adipokine pathways represents a potential area of investigation, but its efficacy and effects on gastrointestinal motility require further validation.

Taken together, these observations suggest that the modulation of nitrergic signaling may represent a common integrative mechanism through which endocrine, metabolic, and neuroimmune signals influence enteric neuromuscular functions.

In this context, the enteric nitrergic system emerges as a dynamic interface integrating endocrine, inflammatory, neural, vascular, microbial, and redox-related signals [32,150,165]. Alterations in adipokine profiles may therefore influence whether NO predominantly functions as a physiological inhibitory neurotransmitter or becomes incorporated into inflammatory and oxidative pathways [3,42,48,135]. This concept may help explain the heterogeneity of gastrointestinal manifestations associated with obesity and metabolic disorders, including impaired gastric accommodation, delayed gastric emptying, altered intestinal transit, visceral hypersensitivity, and neuroimmune dysfunction [166,167,168,169,170,171].

All these data indicate that adipokines may contribute to the regulation of nitrergic homeostasis through effects on NOS/NO signaling, NO bioavailability, oxidative stress, and neuroimmune interactions. However, the extent to which they directly regulate the balance of NOS isoforms within the enteric neuromuscular system remains incompletely established and differs substantially among adipokines.

The available gastrointestinal evidence linking leptin, adiponectin, and resistin to NOS/NO signaling is summarized in Table 1.

## 5. Translational Perspectives and Limitations

All of the current evidence supports an adipokine–NOS signaling mechanism operating across multiple organs and systems, likely including the gastrointestinal tract. Alterations in adipokine profiles associated with obesity and metabolic disorders may influence NOS-dependent pathways, but the direct contribution of these changes to human gastrointestinal motor dysfunction remains to be established.

Although existing evidence relies largely on preclinical studies, targeting adipokine–NOS interactions represents a highly promising avenue for future translational research. Lifestyle interventions, weight loss, dietary modification, physical activity, and emerging metabolic therapies can influence adipokine profiles, and metabolic homeostasis; however, there is currently no direct evidence that these interventions improve gastrointestinal motility specifically by normalizing enteric adipokine–NOS signaling. Future studies should determine whether metabolic interventions modify enteric adipokine signaling, NOS isoform activity, NO bioavailability, and objective measures of gastrointestinal motility, and whether changes in these pathways contribute to clinical improvement. Similarly, circulating adipokines may represent candidate biomarkers, but their diagnostic or prognostic utility requires further validation.

Across the current gastrointestinal literature, the evidence linking adipokines to NOS/NO signaling derives almost entirely from *ex vivo* rodent preparations, predominantly from mouse gastrointestinal preparations generated within a limited experimental literature. Human enteric evidence is nearly absent. These studies provide evidence for pharmacological or molecular associations and, in specific instances, support a functional involvement of NOS in acute gastrointestinal motor responses; however, they do not establish whether alterations in circulating adipokines are primary causes of gastrointestinal dysmotility in humans. In particular, direct enteric leptin–NOS regulation has not been demonstrated, and the proposed consequences of chronic resistin exposure in the gastrointestinal tract remain inferential. Proving causality will require complementary experimental strategies, including intact animal models with selective manipulation of adipokines and their receptors, assessment of NOS isoforms and NO signaling within defined gastrointestinal compartments, and human studies correlating circulating or tissue adipokine levels with objective measures of gastrointestinal motility.

Despite these limitations, adipokine–NOS/NO interactions may represent a biologically relevant area for future translational investigation.

## 6. Conclusions

Accumulating evidence supports adipokines as key modulators of NOS/NO signaling across multiple tissues; however, the extent to which they act as upstream regulators of NOS isoform balance in the gastrointestinal tract remains an open question. Although leptin, adiponectin, and resistin display distinct tissue-specific signaling patterns, direct gastrointestinal evidence is strongest for adiponectin and for the acute modulatory effects of resistin in the mouse gastric fundus.

Current findings support a proposed model in which the adipokine imbalance associated with obesity and metabolic dysfunction may influence constitutive and inducible NOS pathways. However, further validation in human gastrointestinal physiology and disease is required before this model can be considered a causal explanation for dysmotility.

Thus, the adipokine–NOS–motility axis should currently be regarded as an emerging and biologically plausible framework rather than a fully established causal mechanism of gastrointestinal dysmotility. At present, its principal value lies in bridging metabolic, inflammatory, redox, neural, and gastrointestinal observations, thereby identifying specific questions that require direct validation within defined gastrointestinal compartments and in human diseases.

Future efforts should therefore prioritize human tissue characterization, the assessment of adipokine and NOS pathways alongside objective motility measurements, longitudinal studies, and, ultimately, rigorously designed clinical trials before therapeutic or biomarker translational claims can be advanced.

## Figures and Tables

**Table 1:** Summary of gastrointestinal evidence linking adipokines to NOS/NO signaling and gastrointestinal motor function. The table details the experimental context and gastrointestinal region, distinguishing demonstrated functional or molecular relationships from interpretations and hypotheses.

Adipokine	Receptor/Signaling Pathway	Species/Region	Experimental Setting	NOS Isoform Implicated	Direction of Effect	Motility/Neuromuscular Outcome	Evidence Level	Ref.
**Leptin**	Leptin receptor (LEPR); downstream NOS pathway not directly established	Rat Proximal colon	*Ex vivo* smooth muscle preparations	Nitrergic signaling implicated; specific NOS isoform not established	Modulatory	Leptin altered tone and nerve-stimulation-induced contractile and relaxant responses; effects were compatible with involvement of nitrergic neurons	Functional association; direct NOS regulation not demonstrated	[27]
	LEPR; leptin–GDNF signaling	Rat Jejunum/colon	*In vivo* dietary exposure followed by *ex vivo* organ-bath experiments	Nitrergic innervation; specific NOS isoform not established	Context-dependent	Kaolinite-induced changes in enteric responses were associated with altered leptin-sensitive responses; differences were modified in the presence of L-NNA	Functional association; mechanical interpretation	[83]
	Leptin–GDNF signaling	Mouse Gastric antrum	*In vivo* and *ex vivo*	nNOS-containing nitrergic neurons; direct leptin→nNOS regulation not demonstrated	Associated with preservation of nitrergic neurons	Diet-induced obesity prevented age-related loss of antral nitrergic neurons and produced a NO-dependent acceleration of gastric emptying; leptin and GDNF increased	Association; indirect evidence	[84]
**Adiponectin**	AdipoR1/AdipoR2; NO-dependent signaling	Mouse Gastric fundus	*Ex vivo* isolated gastric strips	nNOS/NOS-dependent signaling	Modulatory/inhibitory	Reduced neurally induced contractile responses and enhanced neurally induced relaxant responses; effects were absent after NOS inhibition	Direct functional evidence for NOS involvement	[116]
	AdipoR1; AMPK	Mouse Gastric fundus	*Ex vivo* smooth muscle cells/strips	NO/GC pathway implicated	Inhibitory	Hyperpolarization of gastric smooth muscle cells, increased K^+^ currents, and reduced voltage-dependent Ca^2+^ currents	Direct smooth muscle effect; NOS pathway implicated	[120]
	AdipoR1; AMPK	Mouse Gastric fundus	*Ex vivo* gastric smooth muscle preparations	NOS/NO signaling	Inhibitory	Reduced gastric smooth muscle activity and altered excitation–contraction coupling	Direct functional evidence; NOS involvement context-dependent	[121]
	AdipoR1/AdipoR2; nNOS-related signaling	Mouse Gastric fundus	*Ex vivo* gastric fundus preparations	nNOS	Increased nitrergic response/nNOS expression	Enhanced neurally induced nitrergic relaxation and increased nNOS expression and proportion of nNOS-positive myenteric neurons	Direct functional and molecular evidence	[122]
	AdipoR1; NO-dependent pathway	Mouse Ileum	*Ex vivo* ileal preparations	NOS/NO signaling; specific NOS isoform not established	Inhibitory	Reduced basal tension, spontaneous contractility, and EFS-induced excitatory responses; effects were abolished by NOS inhibition	Direct functional evidence; isoform not established	[117]
	AdipoR1/AdipoR2; sphingosine kinase 2/S1P	Mouse Gastric fundus	*Ex vivo* smooth muscle preparations	NOS not directly assessed as primary target	Modulatory	Altered smooth muscle membrane properties and contractile machinery through SK2 activation	Direct smooth-muscle evidence; relationship to NOS remains unresolved	[123]
**Resistin**	Not fully established in gastrointestinal tissue	Mouse Gastric fundus	*Ex vivo*	nNOS	Increased nitrergic component	Reduced neurally induced excitatory responses and enhanced neurally induced inhibitory responses; effects were abolished by L-NNA	Direct functional evidence	[148]

## Data Availability

No new data were created or analyzed in this study. Data sharing is not applicable to this article.

## Abbreviations

The following abbreviations are used in this manuscript:

AdipoR1/2	Adiponectin receptor 1/2
AMPK	AMP-activated protein kinase
ANO1	Anoctamin 1
BH4	Tetrahydrobiopterin
GC	Guanylate cyclase
EFS	Electrical field stimulation
eNOS	Endothelial nitric oxide synthase
GDNF	Glial cell line-derived neurotrophic factor
iNOS	Inducible nitric oxide synthase
ICC	Interstitial cells of Cajal
LEPR	Leptin receptor
L-NNA	N^G^-nitro-L-arginine
nNOS	Neuronal nitric oxide synthase
NO	Nitric oxide
NOS	Nitric oxide synthase
S1P	Sphingosine-1-phosphate
SK2	Sphingosine kinase 2
TRPM7	Transient receptor potential melastatin-type 7
